# Exploring Metabolic Characteristics in Different Geographical Locations and Yields of *Nicotiana tabacum* L. Using Gas Chromatography–Mass Spectrometry Pseudotargeted Metabolomics Combined with Chemometrics

**DOI:** 10.3390/metabo14040176

**Published:** 2024-03-22

**Authors:** Yuan Jing, Wei Chen, Xuebai Qiu, Shuyue Qin, Weichang Gao, Chaochan Li, Wenxuan Quan, Kai Cai

**Affiliations:** 1 Guizhou Provincial Key Laboratory for Information Systems of Mountainous Areas and Protection of Ecological Environment, Guizhou Normal University, Guiyang 550001, China; 21010160508@gznu.edu.cn (Y.J.); 21010160514@gznu.edu.cn (S.Q.); chaochanli@gznu.edu.cn (C.L.); 2 Upland Flue-Cured Tobacco Quality & Ecology Key Laboratory of CNTC, Guizhou Academy of Tobacco Science, Guiyang 550081, China; chenw@gzyks.cn (W.C.); qiuxb@gzyks.cn (X.Q.); gzyksg@gzmu.edu.cn (W.G.)

**Keywords:** tobacco, geographical region, yield, pseudotargeted metabolomics, metabolic pathway

## Abstract

The quality of crops is closely associated with their geographical location and yield, which is reflected in the composition of their metabolites. Hence, we employed GC–MS pseudotargeted metabolomics to investigate the metabolic characteristics of high-, medium-, and low-yield *Nicotiana tabacum* (tobacco) leaves from the Bozhou (sweet honey flavour) and Shuicheng (light flavour) regions of Guizhou Province. A total of 124 metabolites were identified and classified into 22 chemical categories. Principal component analysis revealed that the geographical location exerted a greater influence on the metabolic profiling than the yield. Light-flavoured tobacco exhibited increased levels of sugar metabolism- and glycolysis-related intermediate products (trehalose, glucose-6-phosphate, and fructose-6-phosphate) and a few amino acids (proline and leucine), while sweet honey-flavoured tobacco exhibited increases in the tricarboxylic acid cycle (TCA cycle) and the phenylpropane metabolic pathway (*p*-hydroxybenzoic acid, caffeic acid, and maleic acid). Additionally, metabolite pathway enrichment analysis conducted at different yields and showed that both Shuicheng and Bozhou exhibited changes in six pathways and four of them were the same, mainly C/N metabolism. Metabolic pathway analysis revealed higher levels of intermediates related to glycolysis and sugar, amino acid, and alkaloid metabolism in the high-yield samples, while higher levels of phenylpropane in the low-yield samples. This study demonstrated that GC–MS pseudotargeted metabolomics-based metabolic profiling can be used to effectively discriminate tobacco leaves from different geographical locations and yields, thus facilitating a better understanding of the relationship between metabolites, yield, and geographical location. Consequently, metabolic profiles can serve as valuable indicators for characterizing tobacco yield and geographical location.

## 1. Introduction

Tobacco (*Nicotiana tabacum* L.) is widely distributed in China’s growing regions and serves as an important model plant for studying plant genetics, breeding, and biochemistry [1]. Tobacco leaves contain abundant metabolites, including saccharides, organic acids, alkaloids, and free amino acids, which play important roles in determining the quality and flavour of tobacco [2,3]. These chemical compositions are strongly influenced by environmental conditions and geographical location. Therefore, investigating the geographical location of metabolites will offer novel perspectives on the formation of regional style characteristics [4].

Metabolomics has been extensively used to trace and analyse the quality of agricultural products from various geographical locations; for instance, previous studies have investigated the bioactive components present in *Glycyrrhiza uralensis* taproots from different locations. Glycycoumarin and licoricone were found predominantly in Jiuquan, while neoliquiritin, isolicoflavonol, isoisoflavone alcohol, and glycerol were mainly detected in Lanzhou [5]. Similarly, Zhao et al. identified 43 differentially expressed metabolites, such as fructose, glycine, and serine, between tobacco leaves originating from Guizhou Province and those from Yunnan Province. These metabolites exert a substantial impact on the tobacco leaf flavour [6]. Guizhou tobacco exhibits distinct characteristics in different geographical regions, such as sweet honey, light, and burnt sweet flavours [7], and distinct metabolic profiles may be observed for different flavour types. Therefore, it is imperative to investigate the relationship between biochemical components and flavour types via metabolomics.

Yield, as an important evaluation index of crops, is also closely related to metabolites. During the formation process, crop yield is affected by the synthesis and degradation of metabolites, such as carbohydrates, proteins, and fats [8]. Many studies have proposed improving plant productivity and yield by increasing the photosynthetic rate and capacity [9,10]. The enhancement of rice productivity and stress resistance under favourable moisture conditions has been demonstrated through the regulation of sugar transport and metabolism, as well as the improvement in photosynthetic capacity associated with high-yield rice gene expression, resulting in a remarkable 30% increase in grain yield [11]. Previous research has found a close correlation between carbon, nitrogen metabolism systems and growth, yield [12]. Therefore, C/N metabolic pathways are intricately associated with plant yield. Significant variations were observed in the phenotypes of tobacco leaves with different yields within the same geographical region. Compared with those of low-yield tobacco plants, the leaves of high-yield tobacco plants are broader and thicker [13]. Consequently, differences in yield inevitably lead to the redistribution of metabolites, causing changes in metabolic pathways [14]. However, studies on metabolic alterations in varying yields are limited. By investigating the metabolic disparities among high-, medium-, and low-yield tobacco leaves, we can identify distinct profiles, as well as biomarkers, that influence metabolic pathways and unravel the correlation between yields and metabolic networks.

In recent years, pseudotargeted metabolomics has emerged as a pivotal tool for investigating plant disease resistance and cultivating superior varieties [15,16]. This technology is designed to rapidly, reliably, and sensitively conduct systematic and comprehensive analyses of characteristic metabolites produced in organisms, tissues, cells, and other systems by monitoring the dynamic changes in plant metabolites and their metabolic pathways [17]. The primary analytical platforms for pseudotargeted metabolomics include gas chromatography–mass spectrometry (GC–MS), liquid chromatography–mass spectrometry (LC–MS), and capillary electrophoresis–mass spectrometry (CE–MS) [18]. Among them, GC–MS is the most widely employed owing to its excellent reproducibility, high precision, extensive dynamic range, and mature metabolite database [19,20]. In 2012, Li et al. first proposed the retention time-locking GC–SIM–MS pseudotargeted metabolomics method and applied it to characteristic metabolites in tobacco leaves from different geographical locations [21]. Cai et al. utilized GC–MS pseudotargeted metabolomics to accurately analyse metabolites in *Oryza sativa* soil, and they demonstrated that this approach enhances the specificity, sample throughput, and coverage of the detected metabolites [22]. This method combines the benefits of both targeted and untargeted approaches, providing high sensitivity, precise quantification, and a broad linear range, and represents a promising technique that has been successfully employed for studying metabolic profiling across various tissue samples [23,24]. Therefore, the utilization of pseudotargeted metabolomics enables more accurate and sensitive monitoring of tobacco metabolites from different geographical locations and yields, with better discerning metabolic characteristics.

In this study, pseudotargeted GC–MS metabolomics was used to investigate the effects of the geographical location and yield factors on the metabolic characteristics, aiming to resolve the following issues: 1. interaction effects of geographical location and yield on metabolites in tobacco leaves; 2. influence of different geographical regions on tobacco flavour; and 3. changes in metabolic profiles under different yields.

## 2. Materials and Methods

### 2.1. Chemicals and Reagents

The metabolite standards were purchased from Sigma Aldrich (MO, USA), Tokyo Chemical Industry (Tokyo, Japan), Aladdin (Shanghai, China), J&K Chemicals (Beijing, China), and Toronto Research Chemicals (Toronto, Canada). The extraction solvents methanol and chloroform were obtained from Sinopharm Chemical Reagent (Beijing, China). Methoxyamine hydrochloride (MEOX, ≥98%), N,O-bis(trimethylsilyl)trifluoroacetamide (BSTFA, ≥98%), and anhydrous pyridine (≥99.5%) were used as derivatization reagents and were obtained from Sigma. Internal standards (ISs) of phenyl beta-D-glucopyranoside (TCI, ≥99%), hexanedioic acid (Aladdin, ≥99%), and *L*-norvaline (Aladdin, ≥99%) were used.

### 2.2. Sample Preparation

Guizhou Province is situated in the southwestern region of China and is characterized by a gradual decrease in elevation from west to east and an increase in average annual rainfall from north to south. Bozhou and Shuicheng are the primary tobacco-cultivation areas for sweet honey and light flavours in Guizhou Province, respectively, and exhibit distinct differences in geographical and climatic conditions. Bozhou is located in the middle of Guizhou Province and has a higher temperature, abundant rainfall, and shorter daylight hours, whereas Shuicheng, located in western Guizhou Province, has lower temperatures, less rainfall, and more intense sunlight. The annual ecological factor data for 2021 were provided by the Guizhou Meteorological Bureau (Table S1). Thirty-six fresh flue-cured tobacco samples (cultivar: Yunyan 87) were collected from Bozhou and Shuicheng at three yield levels in 2021. Samples of low-yield (90–110 kg/mu), medium-yield (120–140 kg/mu), and high-yield (150–170 kg/mu) flue-cured tobacco were collected from more than 500 mu of contiguous tobacco fields with six biological duplicates per treatment. During sampling, the middle leaf was identified as the tenth leaf when counting from top to bottom. The base and tip of each leaf were removed, and the middle portion was retained. Subsequently, each leaf was divided into two halves along the main vein boundary, wrapped in tin foil, and flash-frozen in liquid nitrogen. Then, the samples were freeze-dried and ground into a powder at a low temperature. After passing through a 40-mesh sieve, the samples were stored at −80 °C in an ultralow temperature refrigerator. In addition, quality control (QC) samples were obtained by thoroughly blending with the same amount of each sample.

### 2.3. Metabolite Extraction and Derivatization

The leaf powder (50 mg) was added to a 10 mL centrifuge tube, followed by the addition of 40 µL of internal standard solution (hexanedioic acid at a concentration of 10 mg/mL, phenylglucoside at a concentration of 8.04 mg/mL, and *L*-norvaline at a concentration of 4.9 mg/mL in a methanol–water ratio of 1:1, *v*/*v*). Subsequently, 3 mL of the extract solution (methanol–chloroform–water 2.5:1:1, *v*/*v*/*v*) was added. After vortexing for 1 min, ultrasound extraction was performed at 4–10 °C for 40 min, after which the mixture was centrifuged at 3000–5000 rpm for 5 min. Three-hundred microlitres of supernatant were dried under N_2_ flow at room temperature and then further dried completely by adding three-hundred microlitres of dichloromethane.

Following this step, the derivatization reaction was carried out by reacting with a solution containing MEOX/pyridine (40 µL of 25 mg/mL) as an oximation agent (40 °C, 120 min), which protected carbonyl groups and reduced the ring reactions of sugars to minimize isomer formation. Trimethylsilylation was subsequently performed by adding BSTFA reagent containing TMCS (1%) (81 °C, 90 min), after which 90 µL of acetonitrile was added (81 °C, 90 min) to improve the derivatization efficiency of the amino group. Then, the samples were centrifuged at 10,000 rpm for 3 min, and the supernatant was subjected to GC–MS analysis.

### 2.4. GC–MS Pseudotargeted Metabolomics

GC–MS analysis was performed on an Agilent 7890A-5975C instrument (Palo Alto, CA, USA) equipped with a CTC PAL autoinjection system. Separation was achieved utilizing an HP-5 MS (60 m × 250 µm × 0.25 µm film thickness) capillary column. The injector port temperature was maintained at 280 °C, and a sample volume of 1 µL was injected through the autosampler at a split ratio of 1:10. The flow rate of the helium carrier gas remained constant at 1.0 mL/min. A temperature gradient program was employed for the oven, starting at 60 °C for 2 min, followed by an increase of 5 °C/min until reaching and holding at 230 °C for another 5 min; then, it was further increased by 8 °C/min to reach and hold at 290 °C for 21.5 min, for a total run time of 70 min. The ion source and quadrupole temperatures were set to 230 °C and 150 °C, respectively, while the transfer line temperature was maintained at 280 °C. The mass spectrometer was operated in the electron ionization mode (EI) at 70 eV. The full-scan acquisition mode was adopted for identification within the mass range of 45–600 *m*/*z* with a solvent delay time of 11.90 min. Pseudotargeted metabolomics incorporates an algorithm designed to choose ions for selected ion monitoring (SIM) from identified metabolites. The SIM data were acquired based on the published literature [21], and AMDIS software version 2.73 (Automated Mass Spectral Deconvolution and Identification System) was used for the selection of characteristic ions. The detailed peak table is shown in Table S2. The metabolites in the QC sample were identified using a standard mass spectrometry database (NIST14 and Willy08 library), the literature, and the linear retention index (LRI). Hexanedioic acid (10.00 mg/mL), phenyl beta-D-glucopyranoside (8.04 mg/mL), and *L*-norvaline (4.90 mg/mL) were used as ISs for quantification, and the correction factor was F = 1 for relative quantification.

### 2.5. Statistical Analysis

Chemometric analysis included different multivariate data analysis methods, such as principal component analysis (PCA) and partial least-squares discriminant analysis (PLS-DA). Simca software 13.0 (Sartorius, Umeå, Sweden) was utilized to construct these models. Metabolic pathway analysis, a heatmap, and a volcano map analysis were carried out using metware cloud (https://cloud.metware.cn/) accessed on 16 August 2023. To normalize the data, log transformation and Pareto scaling were performed. The screening of highly characteristic metabolites among the samples was conducted according to the standard of Cai et al. [22]. Chromatograms of the QC samples were generated using Origin 2021 software version SR1 (OriginLab Corp., Northampton, MA, USA).

## 3. Results

### 3.1. Metabolite Identification in Tobacco Leaves

**Figure 1:**
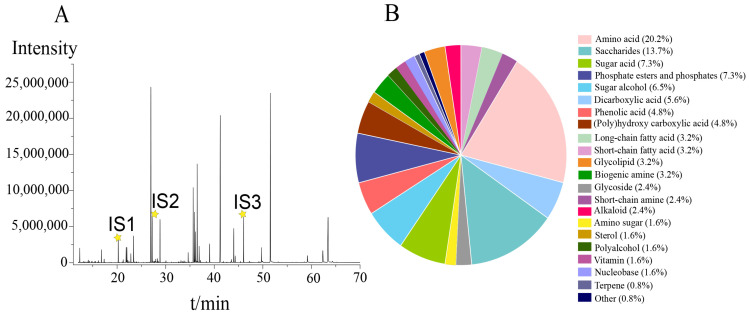
(**A**) Pseudotargeted-mode chromatogram of the QC sample. IS1, IS2, and IS3 represent the chromatographic peaks of the internal standards *L*-norvaline, hexanedioic acid, and phenylglucoside, respectively. 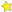 is retention time of IS 1 (20.241 min), IS2 (27.219 min) and IS3 (46.081 min); (**B**) numerical distribution and ratio of 21 chemical classifications.

A total of thirty-six tobacco leaf samples from different geographical locations and yields were comprehensively analysed. Figure 1A shows the typical chromatogram of the QC sample, which represented a ‘mean’ sample containing all possible metabolites. A total of 124 metabolites, mainly amino acids, saccharides, sugar acids, and sugar alcohols, were identified (Table S2). These metabolites were identified with standards and LRIs or mass spectral libraries and were classified into 22 chemical categories (Figure 1B; Table S2). The top ten chemical classifications were amino acids (25 accounting for 20.2%), saccharides (17, 13.7%), sugar acids (9, 7.3%), phosphate esters or phosphate compounds (9, 7.3%), sugar alcohols (8, 6.5%), dicarboxylic acids (7, 5.6%), polyhydroxy carboxylic acids (6, 4.8%), and phenolic acids (6, 4.8%). Short-chain fatty acids, long-chain fatty acids, polyamines, and saccharolactones contributed 3.2% individually. The amino acid group comprised twenty-one proteinogenic amino acids, as well as four nonproteinogenic amino acids or derivatives such as gamma-aminobutyric acid, pyroglutamic acid, 5-hydroxytryptophan, and pipecolinic acid. The saccharide group consisted of thirteen monosaccharides, including hexoses, pentoses, tetrose, and triose, along with four disaccharides. The tobacco pseudotargeted metabolomics approach facilitated the detection of a wider range of metabolites from various chemical classes representing key metabolic pathways for tobacco metabolic profiling.

### 3.2. Interaction Effect of Geographical Location and Yield on Metabolites

**Figure 2:**
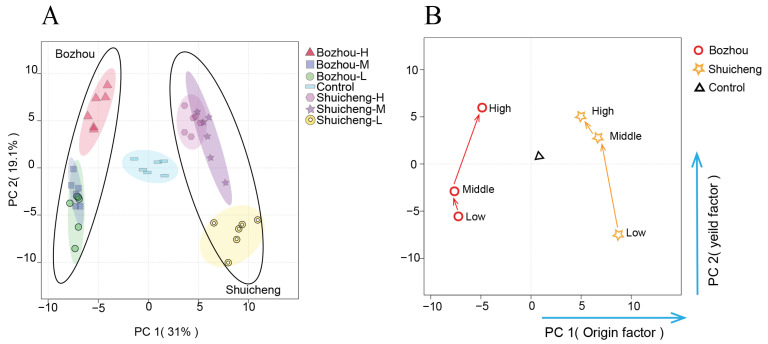
Interaction effect of geographical location and yield on metabolites. (**A**) PCA score plot; (**B**) metabolic trajectory diagram.

PCA was employed to discriminate between the geographical locations of the tobacco leaves from Bozhou and Shuicheng (Figure 2A). The first two principal components (PCs) explained 50.1% of the total variance, with PC1 and PC2 explaining 31.0% and 19.1% of the variance, respectively. PC1 distinguished the geographical location, while PC2 distinguished the yield. The QC samples were tightly clustered in the centre of the score plot, indicating that the sample analysis results were precise. Based on distinct separation, thirty-six samples were categorized into two groups. Significant differences were observed between the Shuicheng and Bozhou regions in PC1. However, samples from the same region, but with different yields, could not be completely distinguished in PC2, such as medium versus low yields in Bozhou and medium versus high yields in Shuicheng. This observation was further supported by the conversion of the PCA data into the corresponding metabolic trajectories (Figure 2B), suggesting that the influence of the geographical location on the metabolite levels may outweigh that of the yield variation.

Based on the loading factor of PCA, the contribution rates of tobacco metabolites to geographical location and yield differentiation were analysed (Table 1). The metabolites that contributed significantly to discriminating the geographical location (absolute value > 0.12) included saccharides, sugar acids, sugar alcohols, and phosphorylated sugars, which indicated that regional factors mainly affected carbohydrate metabolism and phosphorylation. On the other hand, the metabolites that contributed significantly to discriminating the yield factors (absolute value > 0.12) were mainly nitrogenous metabolites, such as amino acids and polyamines, which showed that nitrogen metabolism was a key determinant for achieving the desired crop yields. In brief, the geographical location had a greater influence than yield on metabolic changes in tobacco leaves, and these critical metabolites play crucial roles in plant development and growth regulation.

### 3.3. Metabolic Profiling in Different Geographical Locations

**Figure 3:**
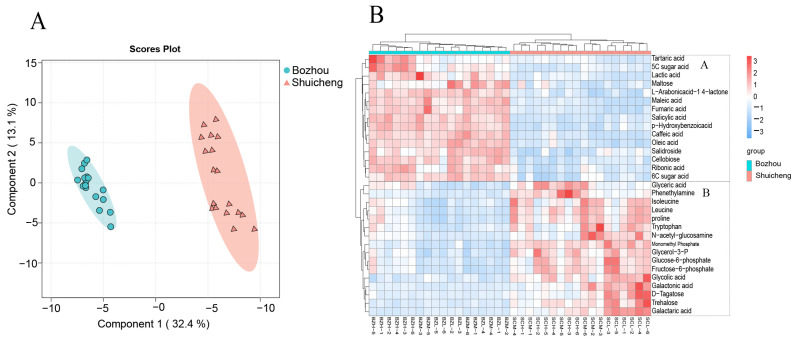
Analysis of metabolites in different geographical locations. (**A**) PLS-DA analysis. (**B**) Heatmap analysis of characteristic metabolites.

To further visualize the differences between the two geographical locations (flavour types) of the metabolites, we applied PLS-DA for discrimination (Figure 3A). The score plots of PC1 and PC2 clearly demonstrated the distinct separation between the Shuicheng and Bozhou samples, accounting for 32.4% and 13.1% of the total variance, respectively. A volcanic map with variable importance in projection (VIP) was drawn according to the screening standards of *p* value < 0.05, FC > 1.5, and VIP > 1.2; thus, 31 characteristic biomarker metabolites were found (Figure S1). These biomarkers included primary metabolites, such as maleic acid, threonic acid, proline, and phenylalanine, as well as secondary metabolites, such as caffeic acid and quinic acid. Subsequently, heatmap analysis was performed on these characteristic metabolites (Figure 3B), which revealed two distinct groups. Group A mainly consisted of the Bozhou samples with greater abundances of phenylpropane metabolism (salicylic acid, VIP = 1.72; p-hydroxybenzoic acid, VIP = 1.7; caffeic acid, VIP = 1.69) and the TCA cycle (fumaric acid, VIP = 1.64; maleic acid, VIP = 1.68), along with sugar acid (6C sugar acid, VIP = 1.58; 5C sugar acid, VIP = 1.32) and saccharolactones (*L*-arabonic acid-1,4-lactone, VIP = 1.66). Group B predominantly comprised the Shuicheng samples exhibiting relatively high levels of sugar metabolism- and glycolysis-related intermediate products (trehalose, VIP = 1.36; glucose-6-phosphate, VIP = 1.3; fructose-6-phosphate, VIP = 1.38) and a few amino acids (proline, VIP = 1.33; leucine, VIP = 1.39), which are all crucial factors influencing the location (flavour type).

### 3.4. Characteristic Metabolites and Their Metabolic Pathways at Different Yields

**Figure 4:**
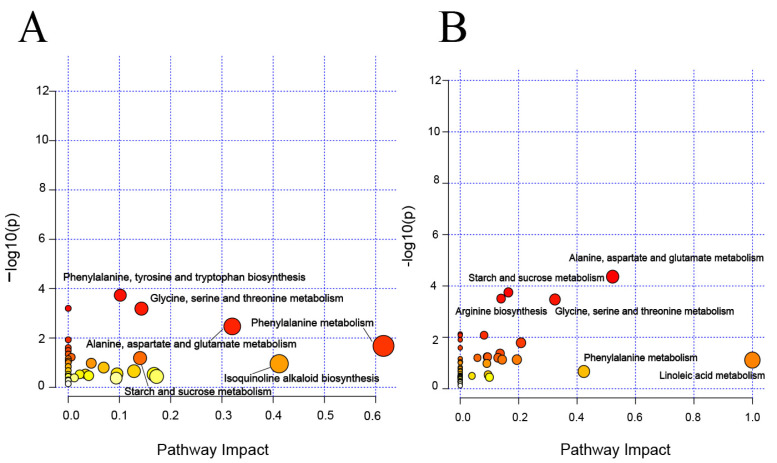
Metabolite pathway enrichment analysis for tobacco leaves with different yields. (**A**) Bozhou. (**B**) Shuicheng. The larger the circle and the darker the colour, the more significantly enriched the metabolites are in this pathway.

The characteristic metabolites in tobacco leaves with different yields were analysed. According to Figure S2, the three yields in the Shuicheng area were effectively distinguished, whereas distinguishing between the middle and low yields in Bozhou was challenging. To further investigate the differences among these three yields in Bozhou and Shuicheng, the PLS-DA of VIP > 1.2, FC > 1.5, and *p* < 0.05 was used to conduct characteristic metabolite screening (Figures S3 and S4), and metabolite pathway enrichment analysis was subsequently conducted at different yields (Figure 4). The main enrichment pathways of the Bozhou samples were phenylalanine metabolism; phenylalanine, tyrosine, and tryptophan biosynthesis; starch and sucrose metabolism; glycine, serine, and threonine metabolism; alanine, aspartate, and glutamate metabolism; and isoquinoline alkaloid biosynthesis (Figure 4A). The main enrichment pathways of the Shuicheng samples were phenylalanine metabolism; arginine biosynthesis; starch and sucrose metabolism; glycine, serine, and threonine metabolism; alanine, aspartic acid, and glutamate metabolism; and linoleic acid metabolism (Figure 4B). These findings suggest that samples from both Shuicheng and Bozhou exhibited changes in six pathways, four of which were the same and mainly involved C/N metabolism.

**Figure 5:**
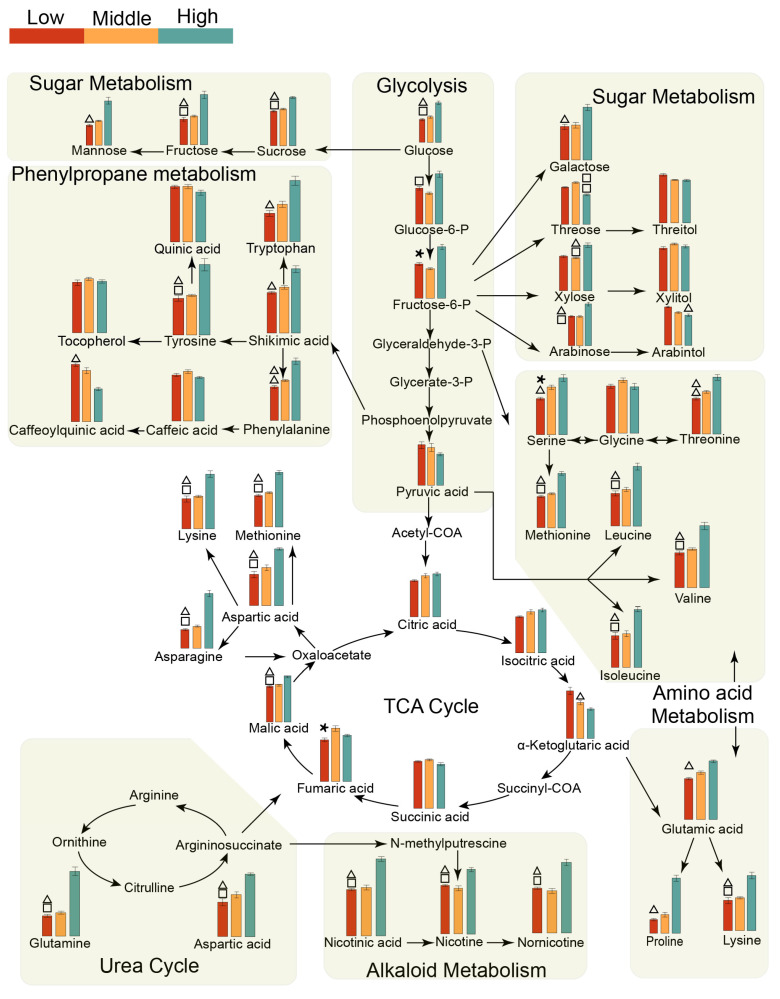
Metabolic pathway plot of the differentially abundant metabolites among the three yields in Bozhou. Red, yellow, and green indicate the relative concentrations of metabolites at low yield, intermediate yield, and high yield, respectively. △, *, and □ represent *p* values of metabolites using nonparametric tests that were less than 0.05 for the low-yield vs. high-yield, low-yield vs. middle-yield, and middle-yield vs. high-yield comparisons, respectively.

Further analysis of these metabolic pathways (Figure 5 and Figure S5) revealed higher levels of intermediates related to glycolysis and sugar metabolism (e.g., glucose, sucrose, fructose, fructose-6-phosphate, and glucose-6-phosphate) in the high-yield tobacco samples than in the other samples. However, sugar alcohols exhibited a significant increase in both the middle-yield and low-yield tobacco samples in the two regions. The content of amino acid metabolism intermediates, such as serine, threonine, and valine, increased in high-yield tobacco leaves. Moreover, the levels of phenylalanine, tryptophan, and shikimic acid were also elevated, indicating that the metabolic pathway of phenylpropane in the high-yield tobacco leaves of the two regions improved. High-yield tobacco leaves also improved the urea cycle in both regions, thereby increasing the content of polyamines and nicotine alkaloids.

## 4. Discussion

### 4.1. Metabolite Identification and Method Evaluation

In general, methanol–chloroform–water is an effective metabolite extraction system for water-soluble and hydrophobic metabolites in plant matrices [23]. The accurate identification of 124 metabolites belonging to 22 chemical categories was successfully achieved in tobacco leaves. These metabolites play crucial roles as significant contributors to the C/N metabolic cycle. In addition, the reproducibility of the results is also a crucial aspect when evaluating the quality of an analytical method [25,26]. All metabolites were subjected to normalization using an internal standard for relative quantification. As indicated in Table S2, 97.6% and 87.9% of all metabolites had a relative standard deviation (RSD) below 20% for repeatability and reproducibility, respectively. The repeatability and reproducibility were considered acceptable and in line with the values commonly found for plant metabolomics (ca. 25–35%) [27]. All the results indicated that tobacco pseudotargeted GC–MS analysis is a dependable approach for metabolic profiling.

Although the precision met the requirements for relative quantification, it is recommended that an appropriate internal standard system with each chemical classification is needed to effectively improve the accuracy and precision. The isotopic or homologous internal standard is the best choice for further research [22]. Furthermore, pseudotargeted analysis cannot detect metabolites that have not been identified. Untargeted metabolomics is a complementary approach for discovering crucial signals of unknown metabolites in tobacco.

### 4.2. Characteristic Metabolites of Different Geographical Locations and Their Effects on Flavour Type

Previous studies have shown that light-flavoured tobacco is characterized by freshness, floral notes, and acidity, while fully flavoured tobacco predominantly possesses a high aroma profile with a rich and pure fragrance [28,29]. Carbohydrates constitute the most significant precursors of aroma in tobacco, accounting for 40–50% of its weight [30,31]. These compounds generate flavour components and acidic substances in mainstream smoke that mitigate the harsh taste during smoking while enhancing the overall flavour characteristics and aroma perception [32]. By screening the characteristic metabolites of the two geographical locations, the abundances of saccharides and phosphorylated sugars in the Shuicheng sample were greater than those in the Bozhou samples. Notably, trehalose, fructose-6-phosphate, and glucose-6-phosphate were identified, two of which are intermediate products of glycolysis (the oxidation process from glucose to pyruvate). Additionally, proline and leucine were more abundant in the Shuicheng samples than in the Bozhou samples; proline contributes to freshness and floral attributes, while leucine significantly enhances acidic notes [33]. Therefore, these metabolic characteristics may lead to the formation of light-flavoured tobacco leaves. It is widely recognized that a decreased nitrogen nutrition level promotes the formation of a delicate aroma profile in flue-cured tobacco, whereas an increased nitrogen nutrition level enhances the expression of a strong and abundant aroma style in tobacco [34]. The abundance of organic acids in the Bozhou samples was much greater than that in the Shuicheng samples. Organic acids play a crucial role in smoke equilibrium and tobacco pH regulation, ultimately influencing the aroma quality indirectly [35]. For instance, Bozhou has a higher concentration of oleic acid than Shuicheng, while high levels of unsaturated fatty acids can enhance the flavour of acidic wax and fat [34]. Moreover, as intermediate products of the phenylpropane metabolic pathway (caffeic acid), the abundance of the Bozhou samples was also greater than that of the Shuicheng samples. Phenylpropanoid biosynthesis in most plants initiates the conversion of phenylalanine to cinnamic acid, resulting in diverse aromatic compounds and affecting the aroma of tobacco leaves [36]. Furthermore, a high concentration of ester compounds leads to a stronger irritant taste, and the *L*-arabonic acid-1,4-lactone in Bozhou samples may be one of the reasons for the abundant aroma [37]. Therefore, these metabolic characteristics may lead to the sweet honey and light flavour types in Guizhou.

### 4.3. Characteristic Metabolites and Metabolic Pathways of Different Yields

The metabolic pathways involved in C metabolism, such as sugar metabolism, glycolysis, the TCA cycle, and shikimate–phenylpropanoid metabolism, play crucial roles in generating energy that can be utilized by plants for growth and development, simultaneously improving the resistance of plants and providing the carbon skeletons necessary for various biosynthetic processes [38]. It is widely acknowledged that the production of carbohydrates in source organs and their utilization in sink organs are tightly coordinated processes that ultimately determine the yield [39]. Starch and sucrose metabolism comprised the main enrichment metabolic pathway, and there was an increase in the abundance of glucose, fructose, sucrose, fructose-6-phosphate, glucose-6-phosphate, and other saccharides in the high-yield tobacco samples from the two regions. The metabolic pathways of starch and sucrose metabolism are complex biochemical processes that rely on the synergistic action of multiple enzymes [40]. Phenylpropane metabolism intermediates are crucial for plant growth and the long-distance transport of water and nutrients while also aiding plant defence against abiotic and biotic stresses [41,42]. For instance, quinic acid plays a vital role as an antioxidant by protecting enzyme structures within plants [43]. Notably, low-yield tobacco leaves exhibited increases in caffeic acid, caffeoquinic acid, and quinic acid levels, indicating an higher expression in the phenylpropane metabolic pathway. Due to cultivation stress, this pathway can produce abundant antioxidants and protect tobacco plants from the stress effects. Conversely, high-yield tobacco showed a significant increase in phenylalanine but a decrease in phenylpropane metabolites due to potential inhibition of phenylalanine ammonia lyase enzyme activity [44].

The metabolic pathways involved in N metabolism, such as amino acid metabolism, polyamine metabolism, and the urea cycle, serve as crucial physiological mechanisms that regulate the synthesis and decomposition of nitrogen-containing compounds in plants [45]. Amino acids serve as precursors for numerous nitrogen-containing compounds [46]. The content of most amino acids in the Bozhou samples decreased from high yield to middle yield to low yield. Notably, the altered metabolic pathways included alanine, aspartate, and glutamate metabolism and glycine, serine, and threonine metabolism in the two regions. Glycine and serine, which are essential components of photorespiration, contribute to the provision of one-carbon (1-C) units that actively engage in diverse metabolic pathways, such as polyamine metabolism and nucleic acid metabolism [47]. Furthermore, high-yield tobacco in Bozhou significantly enhanced the urea cycle, leading to increased contents of polyamines and nicotine while altering the isoquinoline alkaloid biosynthesis pathway. However, the breeding objective of tobacco has always been to reduce the levels of nicotine and related alkaloids [48]. Furthermore, a previous study indicated that treatment of tobacco plants with polyamine biosynthesis inhibitors can reduce the polyamine content and ameliorate the phenotype [49]. Hence, polyamine and nicotine biosynthesis in tobacco involves complex interactions that affect the quality of tobacco leaves.

To summarize, the variation in plant metabolites is primarily influenced by the geographical location and yield [50]. Xu et al. reveal that metabolic differences of E. *purpurea* were related to geographical location (latitude and longitude) and environmental variables (climate and soil) with NMR [51], while, Benmahieddine et al. used HPLC-DA to identify metabolic characteristics of *Pistacia atlantica* Desf. with gender, organ type (roots, buds, and fruits), geographical location, and stage of ripening [52]. The factors influencing metabolites are highly complex. Therefore, Further research needs to consider the effect of more environmental factors and different harvest time on metabolic characteristics and tobacco flavour types. 

## 5. Conclusions

A total of 124 metabolites were identified in Guizhou tobacco leaves of different geographical locations and yields by GC–MS pseudotargeted metabolomics and were divided into 22 chemical categories. Multifactor analysis revealed that the geographical location had a greater influence on metabolites than the yield factors. A screening of the characteristic metabolites in tobacco leaves from different regions revealed that the levels of sugar metabolism- and glycolysis-related intermediate products and amino acids were greater in the Shuicheng samples (light flavour), and the contents of organic acid, sugar acid, and glycolactone involved in phenylpropane metabolism and the TCA cycle were greater in the Bozhou samples (sweet honey flavour). Metabolic pathway analysis revealed that glycolysis and sugar, amino acid, and alkaloid metabolism were maintained at higher levels in the high-yield samples, while higher expression of phenylpropane metabolism was maintained in the low-yield samples.

## Figures and Tables

**Table 1:** Metabolite contributions to geographical location and yield factors.

Compounds	Contribution to Geographical Location Factor	Compounds	Contribution to Yield Factor
Threitol	0.159	Phenylalanine	0.189
6C sugar acid	0.156	Pyroglutamic acid	0.184
*L*-rhamnose	0.154	Phytol	0.179
Arabitol	0.153	Threonine	0.171
Ribonic acid	0.152	Glutamine	0.170
Beta-sitosterol	0.151	Arabinose	0.169
Erythritol	0.150	Nicotinic acid	0.165
Maltose	0.149	Linoleic acid	0.158
Oleic acid	0.148	Methionine	0.157
Salidroside	0.145	Pipecolinic acid	0.148
5C sugar acid	0.144	Ethanolamine	0.147
Gluconic acid	0.141	Shikimic acid	0.146
Mannitol	0.139	Sucrose	0.145
*L*-xylonic acid-1,4-lactone	0.138	Asparagine	0.144
Xylose	0.135	Tryptophan	0.144
Tocopherol	0.134	Aspartic acid	0.143
myo-Inositol-1-phosphate	0.130	Gluconic acid	0.143
Sorbitol	0.129	Tyrosine	0.141
Caffeic acid	0.127	Tyramine	0.140
Cellobiose	0.126	Citric acid	0.136
Glycerophosphoglycerol	0.125	Lysine	0.132
Glyceric acid	−0.125	Malic acid	0.130
Monomethyl phosphate	−0.124	5-Hydroxytryptophan	0.129
*L*-Arabonic acid-1,4-lactone	0.124	Proline	0.128
Fumaric acid	0.123	Xylitol	0.121
Ascorbic acid	−0.122	Tartaric acid	0.120
Sitosterol	0.121		
Salicylic acid	0.121		

## Data Availability

The datasets used during the current study are available from the corresponding author on reasonable request. Data are not publicly available due to privacy.

## Supplementary Materials

The following supporting information can be downloaded at: https://www.mdpi.com/article/10.3390/metabo14040176/s1. Table S1. Sample information; Table S2. List of 124 identified metabolites from 22 classifications detected with pseudotargeted GC-MS. Figure S1. Characteristic metabolite volcano map; Figure S2. (A) PCA analysis of low-, middle-, and high-yields in Bozhou. (B) PCA analysis of low-, middle-, and high-yields in Shuicheng; Figure S3. Volcanic maps and VIP of characteristic metabolites in Bozhou. (A) High- versus low-yield, (B) middle- versus low-yield, and (C) high- versus middle-yield tobacco leaves; Figure S4. Volcanic maps and VIP of characteristic metabolites in Shuicheng. (A) High- versus low-yield, (B) middle- versus low-yield, and (C) high- versus middle-yield tobacco; Figure S5. Metabolic pathway plot of the differentially abundant metabolites among the three yields in Shuicheng.
