# Supplementary material for: Exploring Metabolic Characteristics in Different Geographical Locations and Yields of Nicotiana tabacum L. Using Gas Chromatography–Mass Spectrometry Pseudotargeted Metabolomics Combined with Chemometrics
Source: Metabolites. 2024 Mar 22;14(4):176. doi: 10.3390/metabo14040176 (PMC11052106; doi:10.3390/metabo14040176)
Supplement: Supplementary file 1 [file metabolites-14-00176-s001.zip › metabolites-2912511-supplementary.pdf]

Table S1. Sample Information.

Type	Sample	Duplicates	yeild	Locations	Latitude and longitude	Altitude(m)	Average annual temperature(°C)	Average annual rainfall (mm)	Average annual humidity (%)	Average annual sunshine(hr)
Light-flavor	Shuicheng-H	6	High-yield	Yanjing, Shuicheng, Liupanshui.	26.4368600, 104.9761170	1800	12.9	144.6	78.75	144.4
	Shuicheng-M	6	Middle-yield							
	Shuicheng-L	6	Low-yield							
Sweet honey-flavor	Bozhou-H	6	High-yield	Yingtaojing, Leshan, Bozhou.	27.725654, 106.927389	993.2	15.3	148.3	78.08	114.1
	Bozhou-M	6	Middle-yield							
	Bozhou-L	6	Low-yield							

Table S2 List of 121 identified metabolites from 21 chemical classifications detected with pseudotargeted GC-MS based metabolomics in tobacco leaves

Metabolites	Chemical classification	RT (min)	Identification	RI	RRI	Percent RI error	Derivatization type	CAS Number	Quantitative ion	Confirmative ion	Intra-day RSD/%	Inter-day RSD/%
Short-chain fatty acid												
Pyruvic acid	Short-chain fatty acid	14.584	Standard	1052	1045	-0.7%	1MEOX;1TMS	127-17-3	174	115;73	13.56	19.88
Lactic acid	Short-chain fatty acid	14.917	Standard	1064	1054	-0.9%	2TMS	50-21-5	117	147;219	10.85	15.16
Glycolic acid	Short-chain fatty acid	15.37	Standard	1078	1073	-0.5%	2TMS	79-14-1	147	177;120	6.58	9.89
4-Hydroxybutanoic acid	Short-chain fatty acid	20.081	—	1239	1230	-0.7%	2TMS	591-81-1	233	204;147	2.54	4.43
Long-chain fatty acid												
Stearic acid	Long-chain fatty acid	43.995	Standard	2248	2247	0.0%	1TMS	57-11-4	341	356;117	5.85	9.12
Oleic acid	Long-chain fatty acid	43.544	Standard	2223	2224	0.0%	1TMS	112-80-1	339	129;264	7.85	10.54
Myristic acid	Long-chain fatty acid	34.834	Standard	1851	1843	-0.4%	1TMS	544-63-8	285	204;117	7.58	10.25
Linoleic acid	Long-chain fatty acid	43.555	Standard	2221.8	2199	-0.5%	1 TMS	60-33-3	335	321;350	6.78	12.35
Short-chain amine												
Ethanolamine	Short-chain amine	21.056	Standard	1275	1275	0.0%	3TMS	141-43-5	174	262;147	3.09	7.03
n-Butylamine	Short-chain amine	16.365	Standard	1112	1115	0.3%	2TMS	109-73-9	174	202;128	13.52	19.58
Isoamylamine	Short-chain amine	17.82	Standard	1162	—	—	2TMS	107-85-7	174	216;142	5.85	8.12
Amino acid												
Glycine	Amino acid	16.684	Standard	1124	1135	1.0%	2TMS	56-40-6	102	147;204	12.58	18.33
Alanine	Amino acid	16.215	Standard	1108	1110	0.2%	2TMS	56-41-7	116	190;147	4.98	9.68
Leucine	Amino acid	21.168	Standard	1279	1279	0.0%	2TMS	61-90-5	158	218;232	11.42	15.27
Valine	Amino acid	19.597	Standard	1223	1225	0.2%	2TMS	72-18-4	144	218;246	9.34	12.45
Isoleucine	Amino acid	21.789	Standard	1302	1302	0.0%	2TMS	73-32-5	158	218;232	8.98	16.48
Proline	Amino acid	21.914	Standard	1306	1304	-0.2%	2TMS	147-85-3	142	216;244	8.56	16.38
Serine	Amino acid	23.629	Standard	1371	1369	-0.1%	3TMS	56-45-1	204	218;278	10.02	14.58
Pipecolinic acid	Amino acid	23.801	Standard	1375	1367	-0.6%	2TMS	4043-87-2	156	230;73	9.23	11.5
Threonine	Amino acid	24.429	Standard	1398	1398	0.0%	3TMS	72-19-5	218	291;117	6.54	13.88

Aspartic acid	Amino acid	27.815	Standard	1532	1530	-0.1%	3TMS	56-84-8	232	218;202	8.58	14.12
Pyroglutamic acid	Amino acid	27.821	Standard	1536	1524	-0.8%	2TMS	98-79-3	156	258;230	14.48	23.57
Gamma-aminobutyric acid	Amino acid	27.95	Standard	1542	1542	0.0%	3TMS	56-12-2	174	304;147	8.79	12.89
Glutamic acid	Amino acid	30.05	Standard	1631	1629	-0.1%	3TMS	56-86-0	246	348;147	7.22	9.68
Phenylalanine	Amino acid	30.31	Standard	1643	1640	-0.2%	2TMS	63-91-2	218	192;266	6.89	9.24
Asparagine	Amino acid	31.309	Standard	1686	1686	0.0%	3TMS	70-47-3	231	188;132	9.86	14.87
Glutamine	Amino acid	33.55	Standard	1786	1771	-0.8%	3TMS	56-85-9	347	245;156	8.57	14.03
Lysine	Amino acid	36.599	Standard	1931	1941	0.5%	3TMS	56-87-1	317	174;230	15.47	21.67
Tyrosine	Amino acid	37.032	Standard	1957	1958	0.1%	3TMS	60-18-4	218	280;354	10.58	13.54
5-Hydroxytryptophan	Amino acid	45.65	—	2328	2460	0.0%	4 TMS	56-69-9	290	202;218	9.14	15.45
Tryptophan	Amino acid	44.022	Standard	2248	2237	0.8%	3 TMS	73-22-3	291	202;218	13.23	16.24
Histidine	Amino acid	36.71	Standard	1941	1941	-0.1%	3 TMS	71-00-1	154	254；356	8.79	11.05
Glutmic acid	Amino acid	30.067	Standard	1630	1629	-0.8%	3 TMS	6899/5/4	246	348；147	9.67	16.23
Hydroxyproline	Amino acid	27.973	Standard	1540	1546	-0.6%	3 TMS	51-35-4	332	140；230	13.45	19.89
Methionine	Amino acid	27.727	Standard	1531	1531	0.0%	2 TMS	63-68-3	176	147；128	15.85	17.28
Ornithine	Amino acid	34.52	Standard	1836	1814	0.1%	4 TMS	70-26-8	142	174；73	6.79	12.35
Dicarboxylic acid												
Oxalic acid	Dicarboxylic acid	17.06	Standard	1137	1133	-0.4%	2TMS	144-62-7	147	190;219	18.58	28.93
Maleic acid	Dicarboxylic acid	22.104	Standard	1313	1310	-0.2%	2TMS	110-16-7	147	245;133	15.85	21.58
Succinic acid	Dicarboxylic acid	22.243	Standard	1317	1318	0.1%	2TMS	110-15-6	247	218;129	13.25	15.49
Fumaric acid	Dicarboxylic acid	23.086	Standard	1349	1346	-0.2%	2TMS	110-17-8	245	147;73	7.12	12.95
Malic acid	Dicarboxylic acid	26.93	Standard	1499	1484	-1.0%	3TMS	6915-15-7	233	245;147	9.86	15.52
2-Ketoglutaric acid	Dicarboxylic acid	29.016	Standard	1586	1589	-0.2%	1 MOX 2 TMS	328-50-7	198	288；304	16.75	18.24
Tartaric acid	Dicarboxylic acid	29.865	Standard	1620	1622	0.0%	5 TMS	526-83-0	292	219;423	6.98	12.34
Saccharides												
Glyceraldehyde	Monosaccharide	19.405	Standard	1217	1207	-0.8%	1MEOX;2TMS	367-47-5	147	103;133	21.58	28.98
Erythrose	Monosaccharide	26.154	Standard	1469	1459	-0.7%	1MEOX;3TMS	583-50-6	205	147;117	4.52	7.58

Xylose	Monosaccharide	31.007/31.164	Standard	1673/1681	1669	-0.2%	1MEOX;4TMS	58-86-6	103	217;307	6.32	8.56
Arabinose	Monosaccharide	31.301	Standard	1686	1680	-0.4%	1MEOX;4TMS	10323-20-3	307	103;231	4.56	6.12
Rhamnose	Monosaccharide	32.595/32.742	Standard	1744/1752	1750	0.3%	1MEOX;4TMS	634-74-2	117	277;147	6.78	4.56
Fructose	Monosaccharide	35.912/36.122	Standard	1904/1914	1925	0.6%	1MEOX;5TMS	57-48-7	307	217;277	8.47	10.47
Mannose	Monosaccharide	36.21/36.573	Standard	1918/1936	1900	-0.9%	1MEOX;5TMS	3458-28-4	319	205;217	3.85	6.47
Glucose	Monosaccharide	36.482/36.889	Standard	1931/1951	1926/1937	-0.3%/-0.7%	1MEOX;5TMS	50-99-7	319	205;160	3.71	4.9
Galactose	Monosaccharide	36.385/36.816	Standard	1925/1947	1945	1.0%	1MEOX;5TMS	59-23-4	319	205;347	3.46	5.34
Threose	Monosaccharide	25.448	Standard	1442	1455	-0.9%	1 MOX 3 TMS	29884-64-8	205	147;133	7.26	9.35
D-Tagatose	Monosaccharide	35.051	Standard	1858	1853	1.0%	5 TMS	87-81-0	307	217;277	5.24	12.19
Talose	Monosaccharide	41.899	Standard	1892	1886	0.0%	MEOX;5TMS	2595-98-4	319	147;205	3.23	6.65
Gulose	Monosaccharide	42.634	Standard	—	—	0.1%	—	4205-23-6	319	205;217	4.32	9.87
Sucrose	Disaccharide	51.503	Standard	2707	2714	0.3%	8TMS	57-50-1	361	437;451	4.99	6.23
Trehalose	Disaccharide	53.301	Standard	2800	2816	0.6%	8TMS	99-20-7	361	217;191	1.75	4.02
Maltose	Disaccharide	53.45;54.132	Standard	2803/2836	—	—	1MEOX;8TMS	69-79-4	361	191;281	6.61	20.29
Cellobiose	Disaccharide	53.072	Standard	2745	2758	0.2%	8 TMS	528-50-7	204	361;217	7.75	15.45
**Glycoside**												
Glyceryl-glycoside	Glycoside	44.471	—	2269	2291	1.0%	6TMS	22160-26-5	204	217;351	17.75	21.43
Myo-Inositol-β-D-galactoside	Glycoside	59.163	—	3071	3063	0.2%	9TMS	3687-64-7	204	217;366	16.26	18.49
Salidroside	Glycoside	53.226	Standard	—	—	0.1%	—	10338-51-9	193	361;204	11.67	18.28
**Amino sugar**												
N-acetyl-glucosamine	Amino sugar	40.88	Standard	2120	2137	0.8%	4TMS	7512-17-6	319	205;147	13.07	16.72
Alpha-D-glucosamine	Amino sugar	36.805	Standard	1946.5	1960	-1.0%	6 TMS	66-84-2	203	217;304	15.43	17.28
**Sugar acid**												

Galactonic acid	Sugar acid	38.665	Standard	2030	2039	0.4%	6TMS	576-36-3	217	147;292	18.78	23.75
Gluconic acid	Sugar acid	38.878	Standard	2039	2037	-0.1%	6TMS	526-95-4	333	217;292	12.12	14.23
Galactaric acid	Sugar acid	39.984	Standard	2084	2073	0.6%	6 TMS	526-99-8	333	292;423	6.78	9.46
Glucaric acid	Sugar acid	34.597	Standard	1839	1844	0.0%	6 TMS	87-73-0	333	292;305	8.67	6.78
Threonic acid	Sugar acid	28.85	Standard	1579	1545	0.0%	4 TMS	7306-96-9	292	205;220	7.25	12.778
5C sugar acid	Sugar acid	33.386	—	1781	-—	0.1%	5 TMS	—	292	217;147	4.67	8.25
6C sugar acid	Sugar acid	39.253	—	2053	-—	-0.2%	6 TMS	—	292	217；333	6.67	10.25
6C sugar acid1	Sugar acid	38.773	—	—	—-	0.3%	—	—	292	217；333	11.25	13.26
6C sugar acid2	Sugar acid	37.373	—	—	—-	0.8%	6 TMS	—	333	217;205	14.33	16.38

Sugar alcohol

Mannitol	Sugar alcohol	37.173	Standard	1965	1969	0.2%	6TMS	69-65-8	319	205;147	9.01	11.35
Sorbitol	Sugar alcohol	37.347	Standard	1973	1988	0.8%	6TMS	50-70-4	319	217;205	10.56	11.87
Threitol	Sugar alcohol	27.577	—	1523	1529	0.4%	4TMS	7493-90-5	217	189;205	8.52	11.28
Erythritol	Sugar alcohol	27.4	Standard	1520	1525	0.3%	4TMS	149-32-6	147	117;205	5.14	6.36
Xylitol	Sugar alcohol	31.658	Standard	1702	1703	0.1%	5TMS	87-99-0	103	217;307	8.47	10.68
Arabinitol	Sugar alcohol	32.614	Standard	1744	1759	0.9%	5TMS	488-82-4	217	103;307	3.58	5.21
Myo-Inositol	Sugar alcohol	41.249	Standard	2132	2130	-0.1%	6TMS	87-89-8	305	318;265	4.45	7.8
Galactiol	Sugar alcohol	37.7	Standard	1995	—	-0.7%	6 TMS	608-66-2	319	205;244	5.28	6.56

Phenolic acid

Salicylic acid	Phenolic acid	28.735	Standard	1575	1561	-0.9%	2TMS	69-72-7	267	193;223	8.69	13.32
p-Hydroxybenzoic acid	Phenolic acid	30.191	Standard	1637	1636	-0.1%	2TMS	99-96-7	267	223;193	5.98	8.12
Protocatechuic acid	Phenolic acid	34.53	Standard	1835	1835	0.0%	3TMS	99-50-3	193	370;355	5.75	8.42
Caffeic acid	Phenolic acid	41.789	Standard	2152	2152	-0.2%	3 TMS	331-39-5	396	219;381	4.34	6.25
Caffeoylputrescine	Phenolic acid	65.992	Standard	3441	—	0.5%	—	29554-26-5	174	307；523	6.67	9.23
Caffeoylquinic acid	Phenolic acid	62.355	Standard	3255	3140	0.0%	6TMS	1241-87-8	345	307;255	7.48	14.23

Phosphate esters and phosphates

Glycerol-3-phosphate	Phosphate esters	33.457	Standard	1783	1796	0.7%	4TMS	57-03-4	330	299;357	11.68	18.89
Phosphoethanolamine	Phosphate esters	33.902	—	1806	1809	0.2%	4TMS	1071-23-4	299	370;315	23.58	30.89
Glycerophosphoglycerol	Phosphate esters	43.775	Standard	2238	—	—	5TMS	6418-92-4	357	445;503	13.16	19.85
myo-Inositol-1-phosphate	Phosphate esters	59.163	—	3071	3063	0.0%	9TMS	15421-51-9	204	217;366	12.11	16.34
Glucuronic acid	Phosphate esters	47.145	Standard	—	—	0.1%	—	576-37-4	292	305;217	9.89	13.45
Hexophosphate	Phosphate esters	46.801	Standard	2373	2367	0.7%	—		387	471；299	6.45	18.46
Glucose 6-phosphate	Phosphate esters	46.457	—	2373	2375	0.5%	6 TMS	56-73-5	387	315;299	7.12	12.65
Fructose-6-phosphate	Phosphate esters	46.255	—	2360	2362	0.0%	1 MOX 6 TMS	6814-87-5	387	299;217;315	5.56	12.34
Monomethyl phosphate	Phosphate esters	18.45	Standard	1183	1184	0.1%	2TMS		241	256;211	13.16	19.85
(Poly)hydroxy carboxylic acid												
Quinic acid	(Poly)hydroxy carboxylic acid	35.651	Standard	1891	1900	0.5%	5TMS	77-95-2	345	255;435	6.53	24.47
Glyceric acid	(Poly)hydroxy carboxylic acid	22.829	Standard	1341	1344	0.2%	3TMS	473-81-4	189	133;292	8.12	11.59
Ribonic acid	(Poly)hydroxy carboxylic acid	33.28	—	1779	1779	0.0%	5TMS	17812-24-7	292	333;307	11.39	12.69
Shikimic acid	(Poly)hydroxy carboxylic acid	34.253	Standard	1824	1809	-0.8%	4TMS	138-59-0	204	357;255	8.58	10.23
3-Hydroxy glutaric acid	(Poly)hydroxy carboxylic acid	28.978	—	1587	1590	0.8%	3 TMS	533-62-0	129	247;349	6.56	9.78
Isocitric acid/Citric acid	(Poly)hydroxy carboxylic acid	34.635	Standard	1841	1805/ 1803	0.0%	4 TMS	77-92-9	273	347；363	11.25	15.45
Sterol												
Stigmasterol	Sterol	66.78	Standard	3480	3445	-1.0%	1TMS	83-48-7	343	394;484	12.46	19.68
Beta-Sitosterol	Sterol	68.065	Standard	3546	3509	-1.0%	1TMS	83-46-5	255	396;381	15.89	24.85
Biogenic amine												
Putrescine	Biogenic amine	32.81	Standard	1757	1739	-1.0%	4TMS	110-60-1	174	214;200	10.85	24.25
Tyramine	Biogenic amine	36.464	Standard	1930.5	1933	0.0%	2 TMS	60-19-5	338	174;86	14.23	16.55
Phenethylamine	Biogenic amine	28.87	Standard	1580.8	1568	0.5%	2 TMS	64-04-0	174	250;86	10.11	14.23
Isopentylamine	Biogenic amine	17.826	Standard	1161	—	0.0%	2 TMS	107-85-7	174	216;142	5.23	20.56
Polyalcohol												
2,3-Butanediol	Polyalcohol	14.388	—	1045	1040	-0.5%	2TMS	513-85-9	117	147;133	8.56	13.57

Glycerol	Polyalcohol	21.236	Standard	1282	1282	0.0%	3TMS	56-81-5	117	147;205	2.18	6.02
**Vitamin**												
Tocopherol	Vitamin	62.159	Standard	3245	3156	-1.0%	1TMS	1406-18-4	502	237;277	7.56	9.45
Ascorbic acid	Vitamin	35.335	Standard	1875	1946	1.0%	4 TMS	299-36-5	173	157；316	9.23	15.34
**Nucleobase**												
Guanine	Nucleobase	41.727	Standard	2150	2128	-1.0%	3TMS	73-40-7	352	367;73	24.87	32.85
Adenine	Nucleobase	35.556	Standard	1888	1876	0.5%	2 TMS	73-24-5	264	279;192	12.34	18.67
**Terpene**												
Phytol	Terpene	42.521	Standard	2181	2181	0.7%	1 TMS	150-86-7	143	123;353	16.78	20.89
**Other**												
Urea	Other	18.641	Standard	1192	1194	0.2%	3TMS	57-13-6	147	171;261	13.37	17.23
**Glycolactone**												
L-Mannonic acid gama-lactone	glycolactone	37.631	—	1993	2012	0.1%	4 TMS	22430-23-5	217	361;204	19.89	21.23
L-Arabonic acid-1,4-lactone	glycolactone	30.545	—	1653	1665	0.3%	3 TMS	51532-86-6	217	349;364	13.45	18.89
L-Xylonic acid-1,4-lactone	glycolactone	30.461	—	1648	1647	0.0%	3 TMS	68035-75-6	217	349;364	6.89	10.28
Erythrono-1,4-lactone	glycolactone	24.074	—	1388	1427	0.7%	2 TMS	15667-21-7	262	247;147	5.54	9.98
**Alkaloid**												
Nicotinne	alkaloid	23.38	Standard	1361	—	0.5%	—	54-11-5	84	133;162	7.06	11.38
Nornicotine	alkaloid	27.91	Standard	1540	—	0.0%	1 TMS	494-97-3	220	205;142	6.45	8.98
Nicotinic qcid	alkaloid	21.69	Standard	1298	1298	1.0%	1 TMS	59-67-6	180	136;106	10.38	15.35

Standard, mass spectrometry database (NIST14 and Willy08 library), linear retention index; mass spectrometry database (NIST14 and Willy08 library), linear retention index and literatures

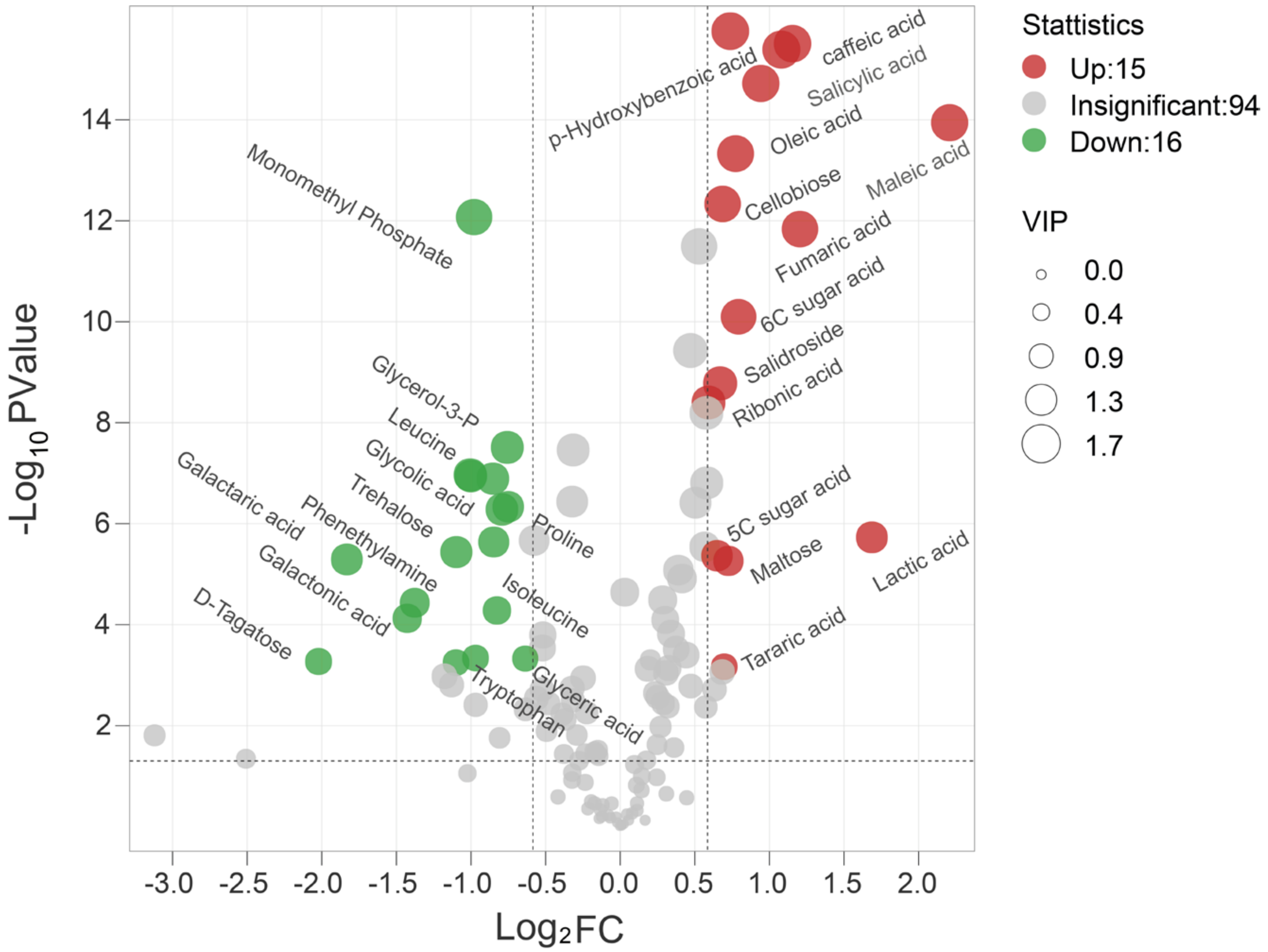

Figure S1. Characteristic Metabolite Volcano Map. P value < 0.05, FC >1.5, VIP >1.2. (Salicylic acid VIP=1.72, p-Hydroxybenzoic acid VIP=1.70, Caffeic acid VIP=1.69, Maleic acid VIP=1.68, Oleic acid VIP=1.68, *L*-arabonic acid-1,4-lactone VIP=1.66 , Fumaric acid VIP=1.64, Cellobiose VIP=1.63, Monomethyl Phosphate VIP=1.62, 6C sugar acid VIP=1.58, Salidroside VIP=1.48, Ribonic acid VIP=1.47, Glycerol-3-P VIP=1.42, Glycolic acid VIP=1.39, Leucine VIP=1.38, fructose-6-phosphate VIP=1.38, Trehalose VIP=1.36, Lactic acid VIP=1.35, Galactaric acid VIP=1.35, Proline VIP=1.33, 5C sugar acid VIP=1.32, glucose-6-phosphate VIP=1.3, Maltose VIP=1.26, Tartaric acid VIP=1.24, Phenethylamine VIP=1.23, Isoleucine VIP=1.22, Galactonic acid VIP=1.22, D-Tagatose VIP=1.21, Tryptophan VIP=1.20, Glyceric acid VIP=1.20).

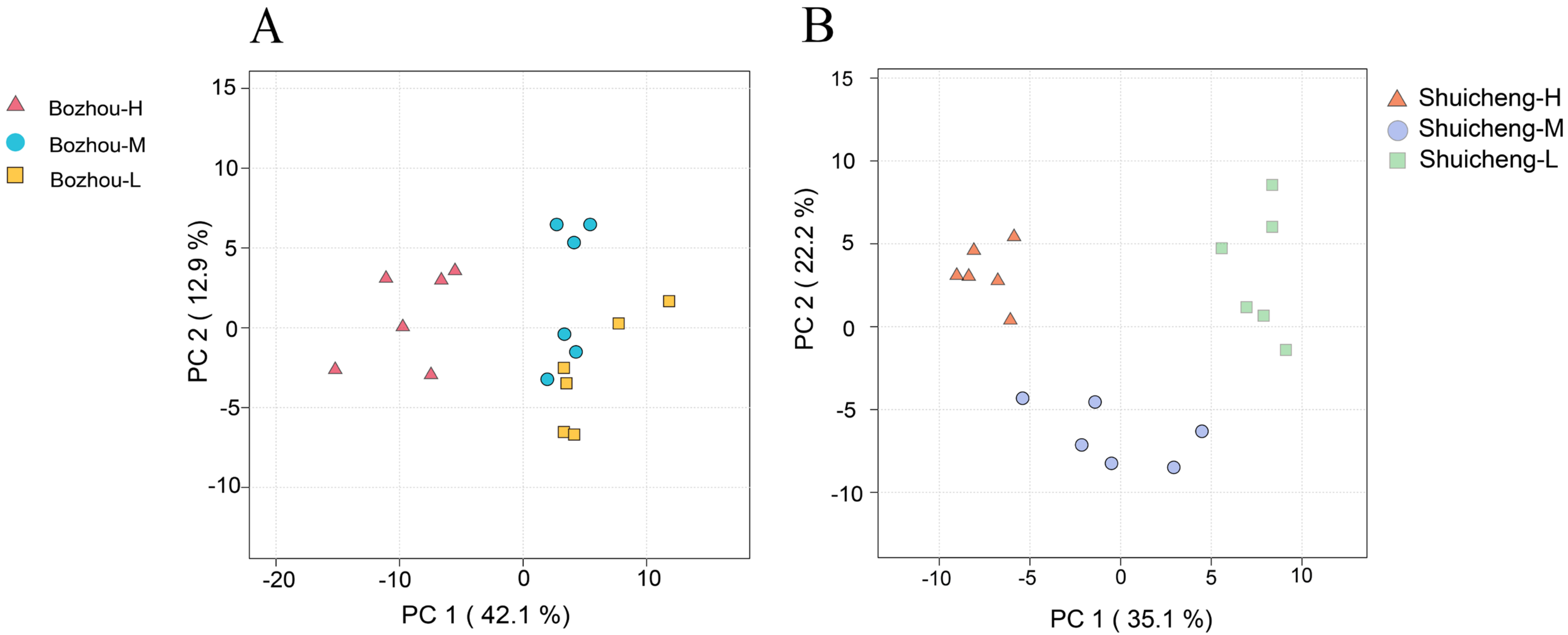

Figure S2. (A) PCA analysis of low-, middle- and high-yield in Bozhou. (B) PCA analysis of low-, middle- and high-yield in Shuicheng.

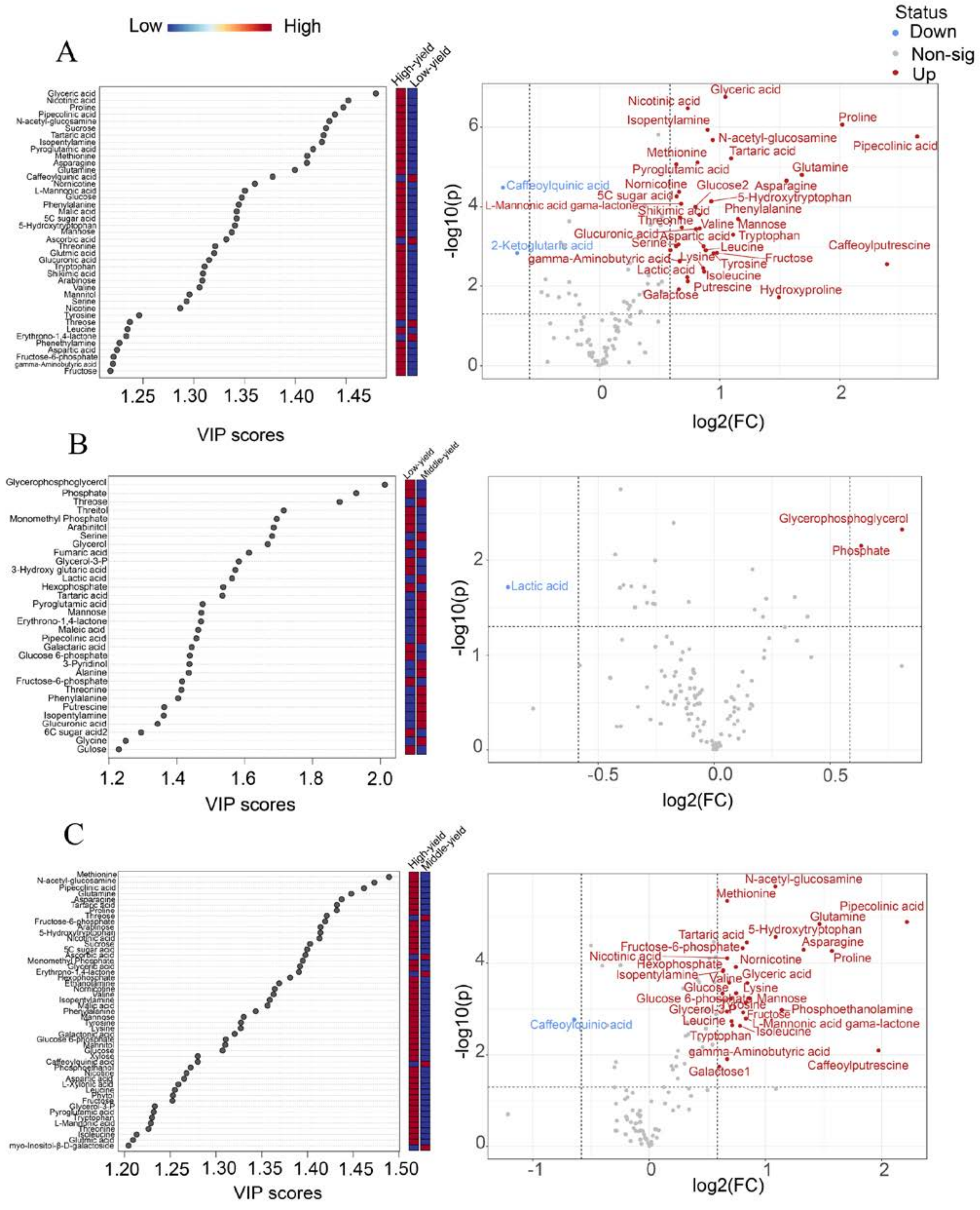

Figure S3. Volcanic maps and VIP of characteristic metabolites in Bozhou. (A) high - versus low-yield tobacco leaves (B) middle - versus low-yield (C) high - versus middle-yield.

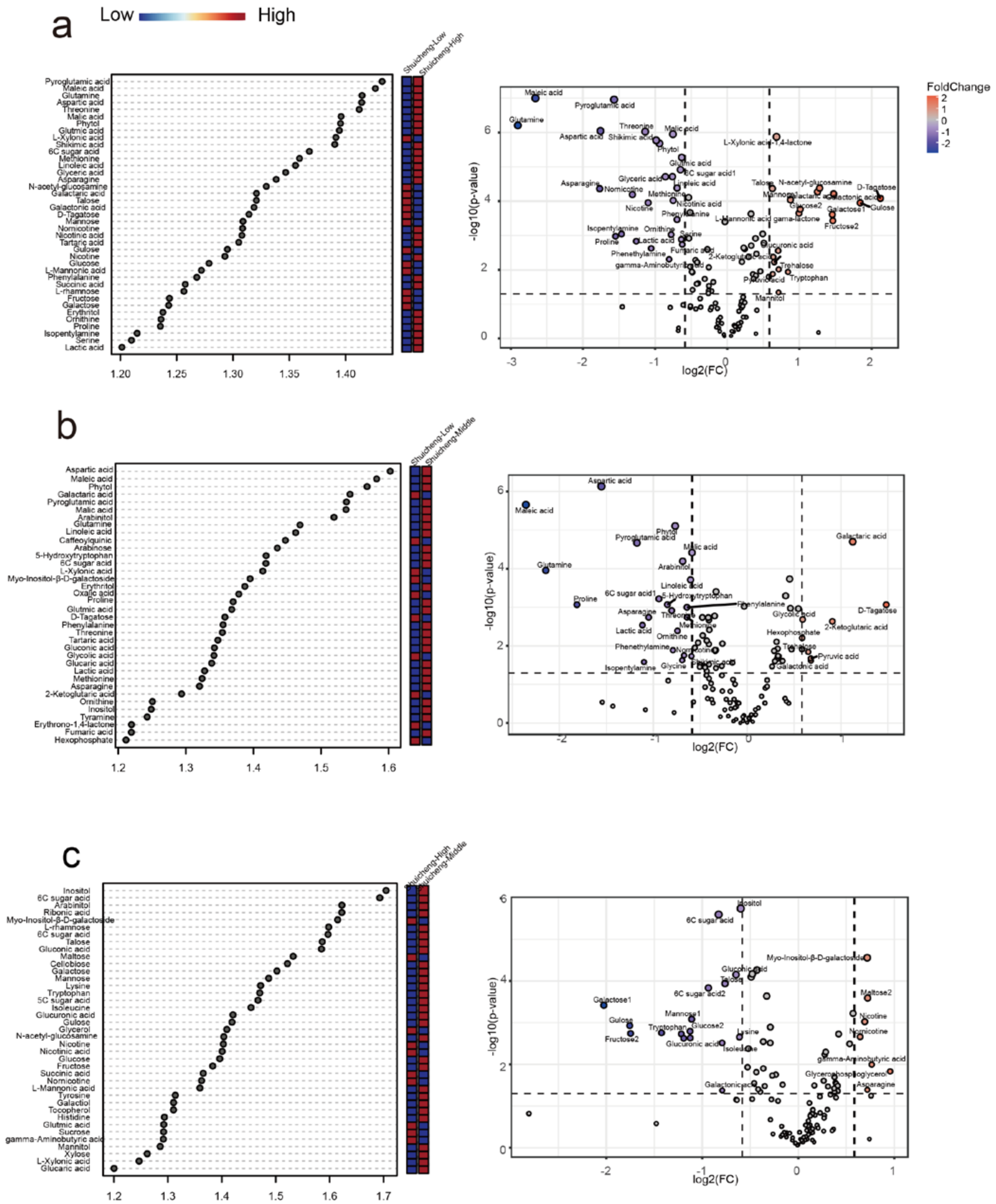

Figure S4. Volcanic maps and VIP of characteristic metabolites in Shuicheng. (A)high - versus low-yield tobacco leaves (B) middle - versus low-yield (C) high - versus middle-yield.

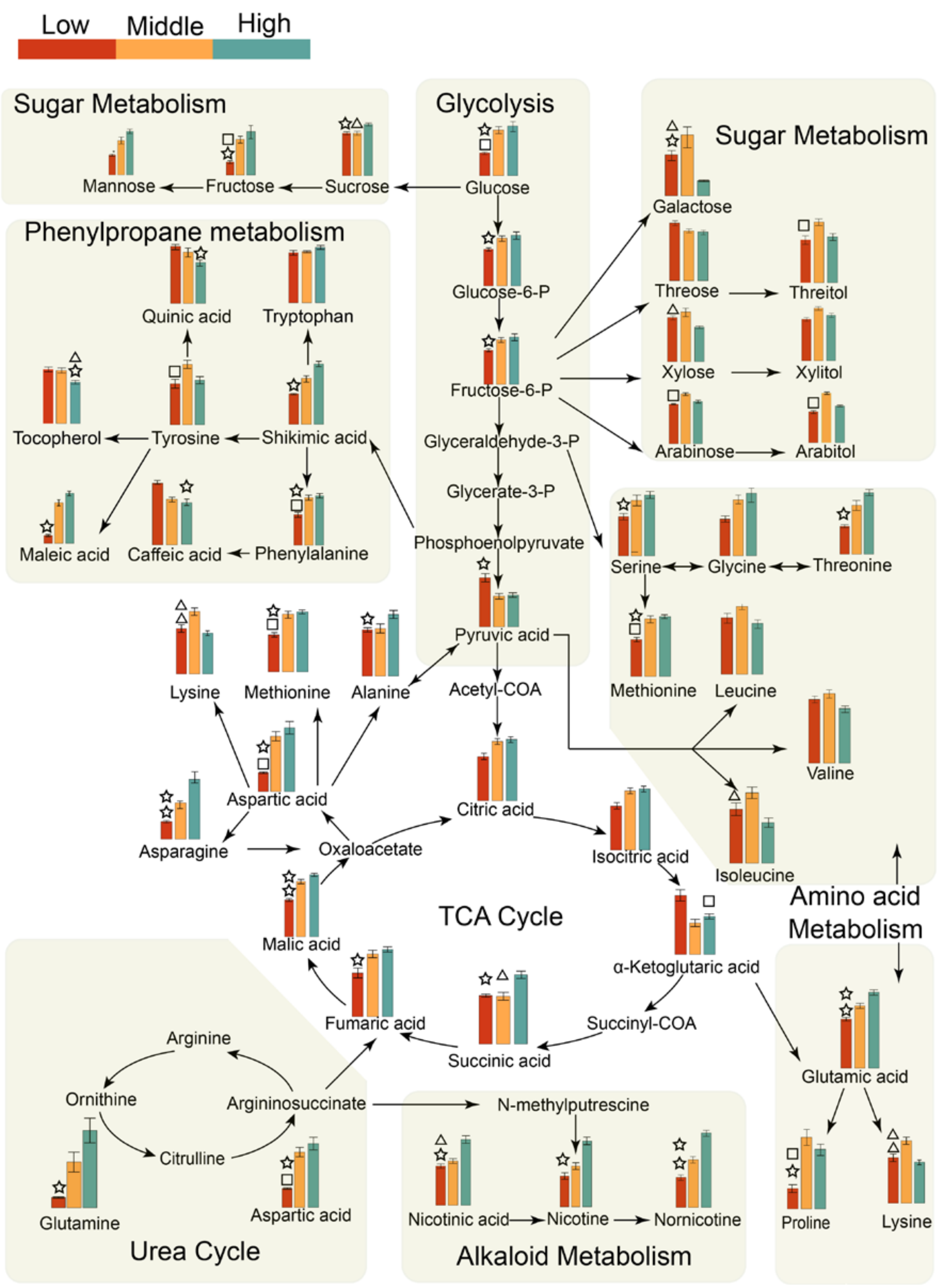

Figure S5. Metabolic pathway plot of the differential metabolites among the three yields in Shuicheng. Red, yellow, and green indicate the relative concentrations of metabolites in the low-yield, middle-yield, and high-yield. △, * and □ represent p values of metabolites using nonparametric tests that were less than 0.05 in low-yield vs high-yield, low-yield vs middle-yield and middle-yield vs high-yield, respectively.
